# Determination of antibiotic residues in frozen poultry by a solid-phase dispersion method using liquid chromatography-triple quadrupole mass spectrometry

**DOI:** 10.1016/j.toxrep.2019.09.005

**Published:** 2019-09-17

**Authors:** Abdulrasaq O. Oyedeji, Titus A.M. Msagati, Akan B. Williams, Nsikak U. Benson

**Affiliations:** a Department of Science Laboratory Technology, The Federal Polytechnic, Ilaro, Nigeria; b College of Science Engineering and Technology, The University of South Africa, Roodepoort, South Africa; c Department of Chemistry, Covenant University, P.M.B. 1023, Ota, Nigeria

**Keywords:** Antibiotic exposure, Antibiotic residues, Mass spectrometry, Frozen poultry, Solid phase extraction

## Abstract

Importation of poultry produce into Nigeria through its land borders has heightened, notwithstanding the government’s ban on such products. This study examined imported frozen poultry products for antibiotic residues considering their health implications. A solid-phase extraction method using liquid chromatography-tandem mass spectrometry in the positive and negative electrospray ionisation and the multiple reaction monitoring modes were employed. The antibiotics were extracted with acetonitrile-dichloromethane. Chromatographic separation was on Waters Acquity UPLC® BEH C18 column with acetonitrile, and water gradient and the antibiotics analysed using Electrospray positive ionisation polarity switch in a single run of fourteen minutes. Residues of nineteen (19) antibiotics were found in the three different matrices at different levels with varying detection frequencies ranging between 2 and 4% (sulfamoxole, penicillin-G, albendazole and phebendazole) and 14–54% for all the other antibiotics. The highest number of violative samples was found in the turkey gizzard and chicken muscle. Sulfixosazole had the highest percentage violation of 80.00% in turkey gizzard while sulfamethoxazole, notwithstanding its lower frequency in chicken muscle had highest maximum concentration and 100% violation. The presence of these drugs, however, does not pose any immediate health risk.

## Highlights

• Twenty-two antibiotic residues quantitatively detected in illegally imported poultry tissues.
• Macrolides, roxithromycin and tylosin were mostly detected in all tissue samples.
• Maximum concentration above MRL recorded for roxithromycin and tylosin with 57.14% and 14.29% violation.
• Sulfonamides indicated high detection frequency in turkey muscle tissues (46.15%).

## Graphical abstract

**Figure:**
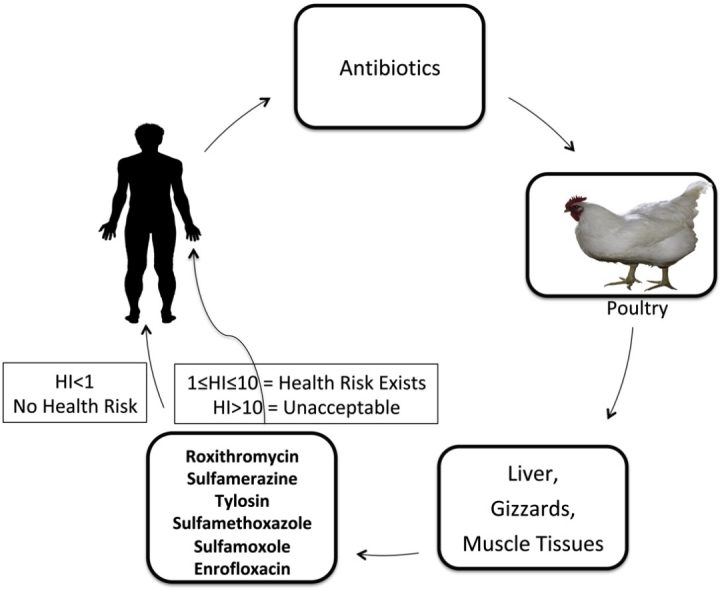

## 1 Introduction

Feeding a global population that will near 10 billion by the year 2050 has led to heavy antibiotic consumption, estimated at 63151 ± 1560 tons in 2010 particularly in poultry production [1]. Nigeria with an estimated 195.9 million people [2], and a growing population projected to surpass the U.S. before the mid-century, has a poultry industry worth $3.5 billion U.S. Dollars. Notwithstanding, poultry products enter into Nigerian markets documented and undocumented, and the business continues to flourish arising from their low cost and citizens low economic power to afford the more expensive domestic poultry meat [3]. Health concerns including antimicrobial resistance arise with over-consumption of products believe to contain antibiotic residues [4]. Antimicrobial resistance is an emerging global threat to public health and the gains made with the discovery of antibiotics, since bacterial resistance genes deriving from animal microbiome can be transferred to human microbiota; [4], and this has been reported in Sub-Sahara Africa [5].

Meanwhile, antibiotic residues in poultry tissues may consist of the original parent compounds as well as its transformation products or conjugates, and possibly all could be present together resulting in direct toxic effect on consumers and allergic reactions in hypersensitive individuals, and above all it has been implicated as one of the leading causes of antimicrobial resistance in human pathogens [6]. The World Health Organisation (WHO) has warned about an imminent antimicrobial resistance crisis, thus putting the antibiotic era at risk if urgent remedial actions are not taken to reduce antibiotic usage in human and veterinary medicine [7].

The antidote, therefore, is to provide reliable data on antibiotic residues in meat products as an avenue to safeguard public health and ensure some level of consumer protection. To our knowledge, no previous studies have attempted the identification and quantification of antibiotics in imported frozen poultry in Nigeria using high-resolution mass spectrometry (HRMS).

The aim of this study, therefore, is to examine these frozen poultry meat products in Ogun State for antibiotic residues using a previously validated extraction technique with liquid-chromatography-triple quadrupole mass spectrometry as there are no known measures in place to guarantee their safety and geographical traceability.

## 2 Materials and methods

### 2.1 Chemicals, reagents and apparatus

Antibiotic standards, Disodium EDTA (OmniPur®), Oxalic acid (ReagentPlus®), Sodium sulphate (≥99%), n-hexane (Emsure®), Dichloromethane (LiChrosolv®), Methanol (LiChrosolv®) were purchased from Sigma-Aldrich (St. Louis, MO, USA). Acetonitrile (≥99.9%) was sourced from Fisher Scientific, Loughborough, Leicestershire, while the SPE columns (200 mg x8 mL tubes) were purchased from Supelco™, Bellefonte, USA. The 0.45 μm GHP ACRODISC 13 mm disposable syringe filter unit was purchased from Agilent, whereas the deionised water (Ultrapure water) used in this study was produced using an Integral 10 Elix Milli-Q system with an LC (Biopak) polisher (Massachusetts, USA). Other apparatuses used include the Waring laboratory blender – Z272205 (Sigma-Aldrich, St. Louis, MO, USA), and the Vortex mixer VM18 (Schiltern Scientific, Beds, UK).

### 2.2 Standard and working solutions

1 mg/mL standard solutions of the different antibiotics were prepared by accurately weighing and dissolving 5 mg standard in 5 mL methanol: water (50:50 v/v) and kept frozen. The working solutions were prepared from the stock by appropriate dilution at the time of use.

### 2.3 Collection of samples

A total of 150 samples of imported frozen poultry produce including turkey muscle, gizzard and chicken muscle were randomly purchased from a list of major markets and supermarkets in different locations of Ogun State, South-Western, Nigeria that shares bother with the Benin Republic. The samples were wrapped in aluminium foil and kept frozen until time of analysis. Sample preparation for UHPLC-QqQ was based on a previous work [8]. Briefly, minced tissues including the muscle and the gizzard (2 g) were separately placed in a 15 mL polypropylene centrifuge tube. Two (2) mL of formic acid (10 mmol/L, in water) was added to the samples and then homogenized for 15 s and 0.8 g sodium sulphate sequentially introduced into the tubes. The samples were shaken vigorously for 1 min followed with the addition of 4 mL of ethyl acetate and then shaken vigorously again for another 1 min. The mixture was centrifuged for 5 min at 4000 rpm, and the supernatant liquid transferred to an SPE column for clean-up. The column was eluted with 2 mL acetonitrile-dichloromethane (50:50, v/v). The eluate, dried at 40 °C under nitrogen flow and re-dissolved with water-methanol (50:50, v/v) solution to a final volume of 1 mL was filtered using a 0.45 μm syringe filter before injection into the LC–MS/MS system. Background interferences were monitored with two samples blank in each batch.

### 2.4 UHPLC information and operating conditions

The column was a Waters Acquity UPLC® BEH C_18_ (2.1 mm x 100 mm, 1.7 μm, particle size). The oven temperature maintained @ 40 °C with a maximum temperature of 90 °C, and mobile phases consisting of water and acetonitrile in gradient fortified with 0.1% formic acid. The injection and rinsing volumes were 10.0 and 500.0 μL, respectively with rinsing speed of 35.0 u L/sec and a rinsing time of 2 s and the runtime was 14 min.

### 2.5 MS/MS conditions

The retention time and MS acquisition parameters, calibration variables, and multiple-reaction monitoring spectra for selected antibiotic residues investigated in the present study are shown in Table 1, Table 2. The probe voltage (kV) was +4.5 and −3.5 for the positive and negative switches, respectively, with a dwell time of 100 ms for each channel and event time of 0.309 s (Table 1). Desolvation line and interface temperature (ºC) were 250 and 350, respectively while nebulizing and drying gas flow (L/Min) were 3.0 and 15.0, respectively, with CID gas pressure at 230 kpa. Peak unification had a separation width of 0.8%, and depth ratio 10.0% and peak integration was done with i-PeakFinder (Algorithm Version1.2).

**Table 1:** Retention time and MS acquisition parameters for the molecular ions.

Antibiotics (R_T_)	[M+H]^+^	Fragment ions (m/z)	Collision Energy (KeV)	Pre-bias (Q1)	Pre-bias (Q3)
Albendazole (7.243)	267.00	235.00 > 192.00 > 160.05	−20.0 > −33.0 > −38.0	−30 > −30 > −30	−24.0 > −18.0 > −29.0
Ampicillin (7.447)	351.10	160.05 > 106.15 > 107.00	−14.0 > −30.0 > −25.0	−30 > −30 > −30	−30.0 > −17.0 > −18.0
Azithromycin (6.076)	749.50	591.30 > 116.10 > 158.10	−32.0 > −48.0 > −42.0	−38 > −40 > −40	−40.0 > −20.0 > −29.0
Ciprofloxacin (3.194)	333.10	315.15 > 232.10 > 289.20	−21.0 > −38.10 > −17.0	−30 > −30 > −30	−22.0 > −21.0 > −29.0
Enrofloxacin (5.926)	360.5	316.2 > 342.15 > 245.15	−21.0 > −21.0 > −27.0	−30 > −10 > −30	−21.0 > −23.0 > −30.0
Erythromycin (6.969)	734.60	158.15 > 576.30 > 83.00	−33.0 > −21.0 > −53.0	−20 > −20 > −20	−29.0 > −40.0 > −14.0
Lincomycin (5.606)	407.50	126.15 > 359.30	−30.0 > −18.0	−30 > −30 > −30	−22.0 > −25.0
Mebendazole (7.419)	297.10	265.05 > 256.20 > 105.05	−21.05 > −6.0 > −35.0	−30 > −30 > −30	−27.0 > −12.0 > −18.0
Penicillin−G (4.856)	367.50	160.0 > 91.10 > 114.10	−16.0 > −48.0 > −36.0	−30 > −30 > −30	−30.0 > −15.0 > −21.0
Phebendazole (8.294)	300	268.00 > 159.0 > 131.05	−21.0 > −37.0 > −50.0	−30 > −30 > −30	−27.0 > −28.0 > −22.0
Roxithromycin (7.298)	837.50	158.05 > 679.40 > 116.10	−38.05 > −24.05 > −47.0	−40 > −40 > −40	−28.0 > −32.0 > −20.0
Sulfadiazine (5.875)	252	156.0 > 157.0 > 93.10	−15.0 > −16.0 > −29.0	−30 > −30 > −30	−28.0 > −29.0 > −16.0
Sulfadimethoxine (7.148)	311.90	156.05 > 157.05 > 108.05	−23.0 > −22.0 > −30.0	−30 > −30 > −30	−28.0 > −30.0 > −20.0
Sulfaguanidine (5.162)	216.0	93.10 > 157.0 > 60.15	−26.0 > −13.0 > −17.0	−30 > −30 > −30	−16.0 > −27.0 > −25.0
Sulfamerazine (6.104)	266.0	156.95 > 93.10 > 64.95	−16.0 > −34.0 > −49.0	−30 > −30 > −30	−29.0 > −17.0 > −24.0
Sulfameter (6.398)	282.0	92.15 > 93.20 > 157.0	−30.0 > −30.0 > −18.0	−30 > −30 > −30	−16.0 > −16.0 > −27.0
Sulfamethazine (6.578)	280.0	187.0 > 186.0 > 125.05	−17.0 > −17.0 > −23.0	−30 > −30 > −30	−19.0 > −19.0 > −22.0
Sulfamethoxazole (6.578)	255.0	93.05 > 157.0 > 92.15	−30.0 > −16.0 > −30.0	−30 > −30 > −30	−16.0 > −30.0 > −16.0
Sulfamoxole (4.696)	269.0	156.90 > 155.95 > 92.05	−16.0 > −15.0 > −30.0	−30 > −30 > −30	−28.0 > −30.0 > 16.0
Sulfaquinoxaline (6.024)	301.90	156.05 > 108.25 > 92.10	−18.0 > −26.0−>−30.0	−30 > −30 > −30	−27.0 > −20.0 > −16.0
Sulfasalazine (7.706)	400.0	382.10 > 224.05 > 118.90	−20.0 > −30.0 > −45.0	−30 > −30 > −30	−26.0 > −22.0 > −22.0
Sulfixosazole (2.639)	269.00	156.95 > 93.00 > 155.90	−13.0 > −27.0 > −14.0	−30 > −30 > −30	−29.0 > 17.0 > −27.0
Tylosin (6.952)	916.50	174.05 > 101.05 > 145.0	−41.0 > −51.0 > −39.0	−26 > −20 > −26	−30.0 > 18.0 > −26.0

**Table 2:** Calibration equation and regression values for the different antibiotics.

Antibiotic	*R*	*R^2^*	Calibration equation	LOD (μg/kg)	LOQ (μg/kg)	% Accuracy
Albendazole	0.9646439	0.9305379	f(x) = 1111.80*x+15558.0	0.91	0.27	107.4
Ampicillin	0.9317763	0.827861	f(x)=−107.523*x+6343.55	10.85	32.88	105.2
Azithromycin	0.9721773	0.9451287	f(x) = 225.101*x+1403.55	0.18	0.53	103.3
Ciprofloxacin	0.9531985	0.9085874	f(x) = 90.0270*x+334.964	0.09	0.28	110.6
Enrofloxacin	0.9625062	0.9264183	f(x) = 125.395*x+4504.46	0.66	1.99	103.1
Erythromycin	0.9584502	0.9186268	f(x) = 1089.58*x+728.707	0.59	1.80	96.6
Lincomycin	0.9169952	0.8408802	f(x) = 5079.21*x+3119.67	0.09	0.28	104.4
Mebendazole	0.9721917	0.9451568	f(x) = 255.366*x+3524.51	2.57	7.63	98.5
Penicillin-G	1.000000	1.000000	f(x) = 137.410*x-294.450	1.87	5.66	94.5
Phebendazole	0.9448609	0.8927621	f(x) = 365.573*x+1481.51	0.21	0.63	114.9
Roxithromycin	0.8998526	0.8097346	f(x) = 1154.43*x+10061.8	0.48	1.44	150.5
Sulfadiazine	0.8987489	0.80775	f(x) = 39.2600*x+961.870	5.48	16.61	147.8
Sulfadimethoxine	1.000000	1.000000	f(x) = 29.4450*x+0	0.85	2.58	94
Sulfaguanidine	0.9498951	0.9023007	f(x) = 55.5947*x+16098.1	1.39	4.21	148
Sulfamerazine	0.9192724	0.8450617	f(x) = 36.3155*x+5962.61	2.49	7.55	117.8
Sulfameter	0.9503192	0.9031066	f(x) = 88.1203*x+1913.21	13.30	40.29	139.3
Sulfamethazine	0.9413978	0.8862298	f(x) = 87.2620*x+804.733	5.19	15.72	111
Sulfamethoxazole	0.8974929	0.8054935	f(x) = 26.0050*x+4541.57	12.36	37.46	144.1
Sulfamoxole	0.9470611	0.8969247	f(x) = 47.0371*x+997.984	2.05	6.17	107
Sulfaquinoxaline	1.000000	1.000000	f(x) = 9.81500*x+0	0.34	1.02	100
Sulfasalaxine	0.9535115	0.9091842	f(x) = 553.022*x+2484.30	0.2	0.6	113.7
Sulfixozazole	1.000000	1.000000	f(x) = 31.4080*x+0	2.58	7.83	112.5
Tylosin	0.9541382	0.9103797	f(x) = 2117.60*x+10825.2	0.15	0.46	99.8

### 2.6 Identification and quantification of antibiotics

The antibiotics were identified when a signal was visible (i.e. signal to noise ratio > 3) for the two transition reactions selected for each analyte, and the retention time of the analyte in the sample extract corresponded to that of the standard with a tolerance of ± 2.5%. Finally, the side-by-side peak option was employed to compare unspiked with spiked samples for specificity and checking any interferences. A 15-point calibration curve was constructed by plotting integrated peak area vs concentration (10–150 μg/kg) for the separate standards. Quantification is achieved using linear calibration curves with very good correlation coefficient (r^2^  ≥ 0.805) (Table 2). Reagent blanks were analysed at regular intervals as part of the method quality assurance to check equipment drift.

### 2.7 Human exposure risk assessment

#### 2.7.1 Estimated daily intake (EDI) and hazard quotient (HQ) analysis

As there are no official data on the daily poultry consumption in the study area, the mean annual poultry intake per person provided by the FAO [3] was used for the calculation of the estimated daily intake (EDI) of antibiotic residues, and consequently the hazard index (HI) for orientation purpose. The EDI was calculated using Eq. (1) [9,10]:

(1) $$\text{E}\text{D}\text{I}\;=\;({\text{Σ}}_{c\;})(C\;N^{-1}D^{-1}K^{-1})$$

Where

${\text{Σ}}_{c}$ = sum of antibiotic residues in the analysed samples (ng/g)

C = the mean annual poultry intake of poultry meat per person (5.39 kg) [3]

N = total number of samples analysed

D = number of days in a year

K = average body weight considered was 70 and 48 kg for an adult and children aged between 6–18 years, respectively [11].

The hazard index (HI) was computed using Eq. (2)

(2) $$\text{H}\text{I}\;=\frac{EDI}{ADI}$$

Where ADI is the acceptable daily intake for veterinary pharmaceuticals (50 μg/kg body weight, upper bound) [12,13].

## 3 Results and discussion

### 3.1 Limit of detection (LOD) and quantification (LOQ)

The limit of detection (LOD) and quantification (LOQ) for the various drugs ranged between 0.09–13.30 μg/kg and 0.27–40.29 μg/kg, respectively. The LOQ was below the maximum residue limit for the various antibiotics, with signal-to-noise ratio of 6.03 – 568. The accuracy for all the determinations ranged between 94.0 and 148.8%.

### 3.2 Levels of antibiotic residues in tissue samples

The occurrence and concentrations of twenty (22) antibiotic residues in imported frozen turkey muscle, gizzard and chicken muscle as detected and quantified by an LC—MS/MS method are shown in Table 3. Residues of nineteen (19) antibiotics were found in the three different matrices at different levels with varying detection frequencies ranging between 2 and 4% (sulfamoxole, penicillin-G, albendazole and Phebendazole) and 14–54% for all the other antibiotics. The sulfonamides excluding sulfamoxole, sulfaguanidine and sulfaquinoxaline have very high detection frequency in frozen turkey muscle, maximum concentration and percentage residue violation ranging between 5 and 46.15%. Frozen turkey gizzard also had all the sulfonamides but with maximum concentration in most instances below the maximum residue limit (MRL) and percentage violation that was limited to sulfadimethoxine (16.67%), sulfamerazine and sulfamethazine, 33.33% each while sulfixosazole had the highest percentage violation of 80.00% in turkey gizzard. Sulfamoxole was not detected in the chicken muscle while the frequency of violation for the other sulfonamides in the chicken muscle was between 4 and 28% with sulfamethoxazole at the lower limit while sulfixosazole and sulfasalazine were at the highest end. Sulfamethoxazole, notwithstanding its lower frequency, had the highest maximum concentration and 100% violation.

**Table 3:** Detection frequencies, contents and violations of antibiotics in frozen turkey muscle, gizzard and chicken muscle.

Antibiotics		FTM (n = 50)				FTG (n = 50)					FCM (n = 50)				
	n (%)^I^	Mean ± SD (μg/kg)	Range (μg/kg)	MC (μg/kg)	N %^ii^	n (%)^i^	Mean ± SD (μg/kg)	Range (μg/kg)	MC (μg/kg)	N %^ii^	n (%)^i^	Mean ± SD (μg/kg)	Range (μg/kg)	MC (μg/kg)	N % ii
**Sulfonamides**															
Sulfamoxole	1(2)	28.71 ± 0.00	0.00	28.71	0	1(2)	61.64 ± 0.00	0	61.64	0	–		–	–	–
Sulfadiazine	11(22)	41.89 ± 4.56	147.15	151.04	18.18	5(10)	41.09 ± 16.00	71.42	78.98	0	3(6)	39.85 ± 38.09	73.56	82.35	0
Sulfadimethoxine	20(40)	32.97 ± 33.52	147.19	148.80	5.00	9(18)	89.14 ± 94.28	313.61	333.61	16.67	9(18)	43.13 ± 60.02	184.22	192.78	11.11
Sulfaguanidine	8(16)	10.58 ± 4.72	13.84	15.96	0	3(6)	6.15 ± 4.31	8.16	9.44	0	6(12)	14.12 ± 10.25	26.42	33.08	0
Sulfamerazine	10(20)	70.97 ± 73.24	225.81	235.06	20.00	6(12)	102.89 ± 63.02	166.40	147.69	33.33	6(12)	35.49 ± 19.90	54.38	66.53	0
Sulfameter	14(28)	42.79 ± 52.49	214.27	218.81	7.14	7(14)	38.62 ± 14.48	43.06	60.20	0	9(18)	36.50 ± 20.06	58.97	70.35	0
Sulfamethazine	17(34)	52.08 ± 33.06	110.68	123.009	5.88	6(12)	84.80 ± 84.00	213.42	237.63	33.33	10(20)	86.15 ± 92.85	313.18	342.00	10.00
Sulfamethoxazole	9(18)	208.31 ± 330.97	1042.36	1083.44	44.44	1(2)	28.22 ± 0.00	0	28.22	0	2(4)	284.94 ± 228.93	323.76	446.81	100.00
Sulfaquinoxaline	13(26)	21.30 ± 18.57	59.09	60.00	0	6(12)	33.95 ± 26.64	54.18	60.00	0	11(22)	44.46 ± 50.53	145.95	147.50	0
Sulfixosazole	26(54)	110.13 ± 173.54	712.94	722.99	46.15	10(20)	128.44 ± 90.46	191.46	231.81	80.00	14(28)	81.78. ± 91.54	269.21	286.74	50.00
Sulfasalazine	20(40)	33.75 ± 50.27	233.78	235.12	5.00	11(22)	21.57 ± 9.60	30.42	39.44	0	14(28)	50.47 ± 66.59	197.72	199.09	14.29

**β-lactams**															
Ampicillin	7(14)	908.20 ± 40.14	2666.12	2667.32	57.14	2(4)	476.42 ± 40.14	56.77	504.81	100	3(6)	201.97 ± 303.75	526.11	552.70	33.33
Penicillin-G	1(2)	11.93 ± 0.00	0.00	11.93	0	–	–	–	–	–	1(2)	5.08 ± 0.00	0	5.08	0

**Fluoroquinolones**															
Ciprofloxacin	8(16)	17.56 ± 25.50	78.13	79.21	0	1(2)	5.25 ± 0.00	0	5.25	0	5(10)	39.23 ± 34.46	79.82	98.76	0

**Lincosamides**															
Lincomycin	11(22)	1.64 ± 0.11	0.34	1.88	0	3(6)	1.58 ± 0.02	0.04	1.59	0	2(4)	1.59 ± 0.10	0.20	1.70	0

**Benzimidazoles**															
Albendazole	2(4)	11.03 ± 6.44	21.45	23.78	0	–	–	–	–	–	–	–	–	–	–
Mebendazole	11(22)	19.70 ± 11.05	35.04	38.14	0	3(6)	16.97 ± 6.48	22.73	24.05	0	13(26)	13.23 ± 9.21	30.09	25.42	0

**Macrolides**															
Azithromycin	6(12)	4.80 ± 3.66	8.57	9.84	0	2(4)	22.96 ± 5.19	7.35	26.63	0	3(6)	17.19 ± 6.55	12.59	24.54	0
Erythromycin	8(16)	5.24 ± 5.88	18.34	18.66	0	2(4)	4.06 ± 1.85	0	5.98	0	2(4)	25.87 ± 26.79	37.88	6.93	0
Phebendazole	2(4)	12.13 ± 0.00	0.00	12.13	0	–	–	–	–	–	–		–	–	–
Roxithromycin	14(28)	131.45 ± 107.86	354.76	358.55	57.14	6(12)	60.89 ± 57.90	115.83	120.47	50	6(12)	47.072 ± 71.54	183.64	191.47	16.67
Tylosin	14(28)	66.09 ± 41.15	141.69	161.84	14.29	6(12)	107.93 ± 50.71	130.09	179.21	50	7(14)	43.49 ± 20.11	47.88	71.17	0

Key: FTM- Frozen turkey muscle, FTG- Frozen turkey gizzard, FCM- Frozen chicken muscle.

i Positive detection (detection frequency, %).

ii Violation (violation frequency, %), MC: Maximum content; - Not detected.

The EU commission regulation number 37/2010 sets the MRL for all sulfonamide analogues at 100 μg/kg for the different tissues, and their sum in a food matrix should not exceed the MRL [14]. This study, however, recorded a very high concentration of sulfonamides in the different matrices considered, and also with very high maximum concentration limits that exceed the regulatory limits. Such concentration of sulfonamides was reported in chicken muscles from Romania at 180–300 μg/kg [15]. Concentration of 1300 and 3600 μg/kg have been reported for sulfamethazine, sulfonamides in two chicken samples from Ho Chi Minh and Nha Trang cities in Vietnam [16], while those from Alfenas, Brazil had no sulfonamides [14]. Sulfadiazine, sulfamethazine, sulfamethoxazole and sulfaquinoxaline in chicken samples from Peninsular Malaysia had a concentration range of 4 −152 μg/kg [17]. The greatest challenge from sulfonamides in foods is drug resistance in human bacterial pathogen derived from mutations in the dihydropteroate synthase gene, which results in enzymes with structural alterations, and they are most favoured among poultry farmers considering their cost [13].

Among the macrolides, roxithromycin and tylosin had the highest frequency occurrence in all the samples. The maximum concentration for roxithromycin and tylosin in the turkey muscle was above the MRL with 57.14 and 14.29% violation for roxithromycin and tylosin, respectively. The other macrolides, including azithromycin, erythromycin and phebendazole had detection frequency that ranged between 4 and 16%. The maximum concentration for azithromycin, erythromycin and phebendazole was below the MRL, and no percentage violation was recorded for the three residues in turkey muscle. Phebendazole was not detected in both turkey gizzard and chicken muscle while azithromycin and erythromycin with lower detection frequency (4%) had maximum concentration that was lower than the MRL and also percentage violation regarding turkey gizzard and chicken muscle. Roxithromycin and tylosin, however, had 12% detection frequency each, and 50% violation in turkey gizzard and chicken muscle.

Macrolides, including tylosin, azithromycin and roxithromycin have been reported in chicken samples from the Hanan region of China. Tylosin at a concentration of 38.752 and 79.211 μg/kg was reported in two chicken samples while azithromycin (27.336 μg/kg) existed in only a sample, and roxithromycin was not detected in all of the samples [18]. Samples from Almeria, Spain had no tylosin [17]. In the present study, the values of these macrolides were generally below the regulatory limit except for roxithromycin and tylosin. Macrolides and their metabolites, when ingested at higher doses, may impair the human vestibule and cochlear nerves, and could also affect the liver and the kidneys, and may lead to an increase in human resistant strains [19].

Two β-lactam residues including ampicillin and penicillin-G were examined in this study. Ampicillin was generally above the permitted level in all the samples as it exceeded the MRL and reaching up to 2667 μg/kg in turkey muscle. The detection frequency for ampicillin in turkey muscle was 14% with 57.14% violation. Turkey gizzard and chicken muscle had 100% violation for ampicillin residue. Penicillin-G occurred in turkey and chicken muscle at low detection frequency, maximum concentration that was below the MRL, and with no violation. Penicillin-G was however not detected in the turkey gizzard samples.

Ampicillin had been confirmed in poultry meat using the four plate test method at 92 100 μg/kg in laying birds meat sampled in Kosice, the Slovak Republic [19]. It was also reported in poultry meat from supermarkets from Gdansk, Poland using capillary electrophoresis with UV detection at 7.6 μg/kg, [20], an amount below the permitted level. However, a related study did not detect this antibiotic residue in chicken samples (including the muscle, liver and gizzard) obtained from supermarkets in Yangzhou, China [21]. Sajjid et al. [22] showed the presence of ampicillin in 4.0% of poultry muscle collected from different locations in Peshawar, Pakistan.

High residue of ampicillin in foods as found in this study could cause the incidence of ampicillin resistant *Enterococcus faecium* from animals in human cohorts, thus increasing the hospital admission and drug failure that result in increased mortality [23]. Ciprofloxacin was confirmed in turkey muscle with detection frequency of 16%, maximum concentration of 79.21 μg/kg that was below the MRL and also zero percent violation. The presence of ciprofloxacin in the turkey gizzard and chicken was also below the MRL and did not violate the regulated level that is allowed.

### 3.3 Estimated daily intake (EDI) and hazard index (HI) associated with exposures

The results of estimated daily intake (EDI) and the hazard index (HI) for the different antibiotics assayed in this study and for the different tissues are as shown in Table 4. The EDI for the different poultry parts was below 1 μg/kg body weight per day, with boiler muscle having the highest value of 0.017 μg/kg bw/day. The hazard index for all the drugs and in all tissues was below 1.

**Table 4:** Estimated daily intake and the hazard index of antibiotics in different poultry tissues.

Poultry		EDI (μg/kg bw*/day	HI
Turkey Muscle	Adult	0.08	1.6 × 10^−3^
	Children	0.08	1.6 × 10^−3^
Turkey Gizzard	Adult	0.03	6.0 × 10^−4^
	Children	0.04	8.0 × 10^−4^
Chicken Muscle	Adult	0.03	6.0 × 10^−4^
	Children	0.04	8.0 × 10^−4^

bw* = body weight; when HI < 1, risk is considered acceptable; when 1≤HI≤10, risk exists, but does not require immediate action; and when HI > 10, the risk is at unacceptable.

The critical assessment of dietary exposure to antibiotics is vital to obtain fundamental data concerning the safety of foods, problems and trends in the intake of the drugs. Notwithstanding the extensive use of antibiotics in the study area, the information pertaining to their daily exposure is not readily available. The estimation of dietary intake of antibiotics was attempted in this study with its attendant hazard index being first of such an attempt in the area under study. The hazard index for all the drugs in all of the tissues was less than 1 (HI < 1), thus the risk associated with the consumption of the poultry products is considered acceptable. However, when 1 ≤ HI ≤ 10, it indicates that risk exists, but does not require immediate action while HI > 10, shows that the risk is at unacceptable [24,25]. Although, the method employed in this study for risk characterisation for a mix of antibiotics residues in foods is generally acceptable for aggregate exposures, other alternative approaches have been proposed and adopted as risk assessment methodologies [25,26].

## 4 Conclusion

This study showed the presence of antibiotic residues in varying degrees in tissue samples of imported frozen chicken commercially sold and consumed by Nigerians. Enhanced concentrations in some tissues may portend long-term health risk to the consumers. However, the calculated risk associated with potential short-term exposure indicated estimates that were below the threshold values of 1. This implies that there might be no potential health risk associated with short-term exposure. However, possible carcinogenic risks could occur due to continuous exposure and bioaccumulation of elevated levels of antibiotic residues. Consumer education and advocacy should be strengthened to increase the awareness of the populace on the dangers inherent in the consumption of unscreened products. Finally, regulatory agencies should be empowered to perform their duties efficiently and ensure that good quality produce are distributed for consumption.

## Declaration of Competing Interest

The authors declare no conflict of interest.
